# FOXM1 promote the growth and metastasis of uveal melanoma cells by regulating CDK2 expression

**DOI:** 10.1007/s10792-024-02943-y

**Published:** 2024-02-11

**Authors:** Xue Bai, Shan Li, Yan Luo

**Affiliations:** Department of Ophthalmology, Guizhou Eye Hospital, The Affiliated Hospital of Zunyi Medical University, Guizhou Branch of National Clinical Research Center for Ophthalmopathy, Special Key Laboratory of Ocular Diseases of Guizhou Province, Zunyi, 563003 China

**Keywords:** UVM, FOXM1, Metastasis, WGCNA

## Abstract

**Background:**

Uveal melanoma (UVM) is an aggressive malignant tumor originating from melanocytes in the eye. Here, we screened the possible genes involved in the development and prognosis of UVM, and identified that FOXM1 and MET were associated with the prognosis of UVM patients. Forkhead box protein M1 (FOXM1) is a transcription factor that regulates the expression of cell cycle-related genes that are necessary for DNA duplication. However, the regulatory mechanism of FOXM1 in UVM was still not clear. Here, we investigated the regulation of FOXM1 in the malignant phenotype of UVM cells and its effect on the prognosis of UVM patients.

**Methods:**

UVM gene expression profiles were obtained using GSE22138 data from the gene expression omnibus (GEO). Weighted gene co-expression network analysis (WGCNA) was used to construct a key module gene for metastasis, which was strongly correlated with UVM prognosis. The latent biological pathways were identified through gene ontology analysis. Protein–protein interaction (PPI) networks and hub shared gene authentication were performed. GEPIA and UALCAN databases were used for the analysis of relationship between candidate genes (FOXM1 or MET) and the prognosis of UVM patients. The abundance of FOXM1 was examined by quantitative real time polymerase chain reaction (qRT-PCR) and western blot. Colony formation and cell counting kit-8 (CCK-8) assays for cell proliferation, wound healing assay for migration, and transwell invasion analysis for invasion were performed.

**Results:**

GEO database showed the differentially expressed genes between UVM samples with or without metastasis, and a key module gene for metastasis was constructed by WGCNA. The PPI network revealed that seven candidate genes (VEGFA, KRAS, MET, SRC, EZR, FOXM1, and CCNB1) were closely associated with UVM metastasis. GEPIA and UALCAN analyzes suggested that FOXM1 and MET are related to the prognosis of patients with UVM. These experimental results suggested that FOXM1 was highly expressed in UVM cells. FOXM1 deficiency represses the proliferative, migratory, and invasive abilities of UVM cells.

**Conclusions:**

FOXM1 silencing may hinder UVM cell progression, providing a novel theoretical basis and new insights for UVM treatment.

**Supplementary Information:**

The online version contains supplementary material available at 10.1007/s10792-024-02943-y.

## Introduction

Melanoma is a cancer of melanocytes that can be divided into multiple subtypes based on body site location, including cutaneous melanoma, uveal melanoma, mucosal melanoma, and acral melanoma [1, 2]. Uveal melanoma (UVM) is an uncommon but highly aggressive intraocular malignancy in adults, which arises from melanocytes [3, 4]. Although it is a relatively rare disease, UVM still has poor prognosis and high recurrence rate [5, 6]. Hence, it is indispensable to further elucidate the pathogenesis of UVM and seek novel therapeutic methods for the systemic treatment of patients with UVM.

The transcription factor forkhead box protein M1 (FOXM1) could play a role in controlling cell multiplication and the repair of fractured DNA strands [7, 8]. A previous study verified that FOXM1 expression is elevated in various human cancers and is associated with tumor development [9, 10]. Additionally, FOXM1 was verified to be related to cutaneous melanoma growth and may serve as a new therapeutic target for cutaneous melanoma [11, 12]. FOXM1 was dramatically overexpressed in UVM tissues analyzed using GSE22138 data from the gene expression omnibus (GEO) public database. Here, we verified the precise function of FOXM1 in UVM pathogenesis. The possible regulatory mechanism of FOXM1 in UVM was further explored.

In this study, the up-regulated genes in UVM tissues were first screened using the GSE22138 database. In addition, the abundance of FOXM1 in UVM cells and the role of FOXM1 in UVM cell vicious behaviors were verified, which may provide a theoretical foundation and novel curative targets for UVM treatment.

## Materials and methods

### Microarray data download and preprocessing

The gene expression profile matrix file from GSE22138 based on the platform GPL570 (including transcription profile data of 63 UVM tissue samples and matching clinically relevant information) was acquired from the gene expression omnibus (GEO) database (https://www.ncbi.nlm.nih.gov/geo/). The grouping was performed based on whether the tumors had metastasis, and the 63 patients were divided into the “No” group (28 cases) and “Yes” group (35 cases).

### Weighted gene co-expression network analysis (WGCNA) and functional annotation

The limma R package (Version 3.38.3; http://www.bioconductor.org/packages/release/bioc/html/limma.html) was utilized to explore the differentially expressed genes between tumor tissues of “No” group (28 cases) and the “Yes” (35 cases), the threshold was set to: |log2FC|> 0 and *P* value < 0.05. The discrepant expression of genes is shown in the form of a heat map and volcano map. Then, the R software package WGCNA was utilized to conduct the weighted analysis of the correlative network of the above-mentioned differential genes. Based on this, the associations among the genes were acquired, and the topological overlap matrix and correlative matrix between the genes were established to estimate the gene network connectivity and identify the soft threshold value. Genes with similar expression levels were divided into one gene module and linked hierarchical clustering was performed. Simultaneously, each module weight in the dataset was assessed, and the dataset with the highest weight was screened for subsequent analysis. The *R* package of WGCNA, its source code, and supplementary materials are available at http://www.genetics.ucla.edu/labs/horvath/CoexpressionNetwork/Rpackages/WGCNA [13]. Subsequently, the modules that were most relevant to metastasis were selected, and gene ontology analysis was performed to make functional annotations for these module genes.

### Screening of metastasis-associated core genes

A protein–protein interaction (PPI) network was constructed by importing the obtained genes into the significant modules into STRING database (https://cn.string-db.org/), and Cyctoscape software (https://cytoscape.org/) was used for visualization. In this setting, the lowest interaction score of proteins > 0.9 was selected as the reliability basis for the interaction between proteins, and out-of-network nodes were hidden. Finally, a network diagram of protein interactions was obtained. Then, PPI Network-related topology analysis was performed using the CytoNCA plugin, taking “Betweenness”, “Closeness”, “Degree”, “Eigenvector”, “LAC,” and “network” as reference standards. The candidate targets were chosen from the genes whose criterion scores were larger than their matching standard mean, and later suffered from the foregoing topological analysis for 2 times to get candidate genes. The relationship between these genes and overall survival was further explored, and genes that were significantly related to prognosis were regarded as core genes.

### Analysis of the prognosis and cancer stages of UVM patients

The relationship between candidate genes (FOXM1 or MET) and the prognosis of UVM patients was analyzed through the online databases GEPIA (http://gepia.cancer-pku.cn/index.html) and UALCAN (https://ualcan.path.uab.edu/analysis.html). 39 UVM patients with high FOXM1 or MET expression and 39 UVM patients with low FOXM1 or MET expression were included in GEPIA database; and 20 UVM patients with high FOXM1 or MET expression and 60 UVM patients with low/medium FOXM1 or MET expression were included in UALCAN database. The Cancer Genome Atlas (TCGA) is a landmark cancer genomics program that sequenced and molecularly characterized over 11,000 cases of primary cancer samples [14]. Here, TCGA database was used to show the expression of FOXM1 or MET in UVM samples based on individual cancer stages (*n* = 39 for stage2, *n* = 36 for stage3 and *n* = 4 for stage4).

### Cell culture

The immortalized human normal epidermal melanocyte cell line (PIG1) and choroidal melanoma cell lines (M619 and MUM-2B) were acquired from Otwo Biotech (Shenzhen, China) and BNCC (Beijing, China). PIG1 and MUM-2B cells were cultured in 90% DMEM (Procell) and M619 cells were propagated in RPMI-1640 medium (Procell, Wuhan, China) in an incubator with 5% CO_2_ at 37 °C. All media were supplemented with 10% FBS (Procell) and 1% antibiotics (Procell).

### Cell transfection

For FOXM1 silencing, FOXM1 small interference RNA (si-FOXM1#1, si-FOXM1#2, and si-FOXM1#3) were generated, and si-NC served as homologous contrasts. RiboFECTTM CP Reagent Ribobio (Guangzhou, China) and the designated oligonucleotides or plasmids from RiboBio were individually transduced into UVM cells.

### Quantitative real-time polymerase chain reaction (qRT-PCR)

Total RNA segregation was performed using Triquick Reagent (Solarbio, Beijing, China). Subsequently, the cDNA of FOXM1 was amplified from RNA using the SweScript RT I First Strand cDNA Synthesis Kit (Servicebio, Wuhan, China), and the synthesized cDNA was used for qRT-PCR with 2 × SYBR Green qPCR Master Mix (Low ROX) (Servicebio) and specific primers on a PCR system. The RNA abundance of FOXM1 was computed using the 2^−ΔΔCt^ strategy and normalized to the level of inner contrast GAPDH. The primer sequences were as follows: FOXM1-F, TCTGCCAATGGCAAGGTCTCCT; FOXM1-R, CTGGATTCGGTCGTTTCTGCTG; GAPDH-F, GTCTCCTCTGACTTCAACAGCG; GAPDH-R, ACCACCCTGTTGC TGTAGCCAA.

### Western blot

The extractive protein was obtained employing RIPA buffer (Solarbio). The protein was segregated using SDS-PAGE gel (10%; Beyotime, Shanghai, China) and subsequently received the transference to PVDF membrane (Beyotime). Following sealing with 5% slim milk in indoor environment for 1 h, the membrane was reacted with primary antibodies against FOXM1 (13147-1-AP, 1:1000, Proteintech, Wuhan, China), CDK2 (10122-1-AP, 1:2000, Proteintech), and internal protein standard GAPDH (1:2000; 10494-1-AP; Proteintech) overnight at 4 °C. Subsequently, Goat Anti-Rabbit IgG H&L secondary antibody (Alexa Fluor® 680) (ab175773, 1:20,000, Abcam, Cambridge, UK) was utilized to interact with the membrane for 2 h in indoor environment. Later, BeyoECL Star Kit (Beyotime) was utilized for visualizing the immunoblots.

### Cell counting kit-8 (CCK-8) assay

To analyze viability, the transduced MUM-2B and M619 cells were cultivated in 96-well plates for specified times (0, 24, 48, or 72 h), and then every well was supplemented with CCK-8 solution (Servicebio) for 4 h at 37 °C. Finally, the OD value was recorded using a microplate reader at a wavelength of 450 nm.

### Wound healing assay

MUM-2B and M619 cells were maintained in 6-well plates to analyze cell migration 24 h after transfection. Later, scarification was performed using aseptic pipette tips (200 μL) on the cell monolayers, and the floating cells were removed with PBS. After 0 or 24 h of cultivation, the images were acquired using a microscope, followed by the analysis of the wound widths (migrated distance) at 40 × magnification using ImageJ software.

### Transwell assay

The invasive abilities of MUM-2B and M619 cells were tested using a transwell chamber (8 μm, Corning, Cambridge, MA, USA), in which the insert membrane was covered with Matrigel (Solarbio), the upper compartment was added to transfected cells in non-serum medium, and the bottom compartment was replenished with 600 μL complete medium containing FBS. After 24 h, the metastasizing cells in the bottom compartment were immobilized and stained with 4% paraformaldehyde and 0.1% crystal violet (Solarbio) for 1 h. Finally, a microscope was used at 100 × magnification to observe and take a picture of the invasive cells from five randomly chosen areas.

### Colony formation assay

MUM-2B and M619 cells were seeded in 6-well plates prior to siRNA transfection and maintained in complete medium for 2 weeks at 37 °C. Next, the cells were rinsed with PBS, fixed, stained using 4% paraformaldehyde and 0.1% crystal violet (all from Solarbio), and photographed using a camera.

### Statistical analysis

All data from the lowest three repeats are displayed as the mean ± standard deviation (SD) and charted using GraphPad Prism 7 software. Differences were compared using Student’s* t*-test or ANOVA. Statistical significance was defined as *P* < 0.05.

## Results

### Identification of differentially expressed genes between UVM samples with or without metastasis

**Fig. 1:**
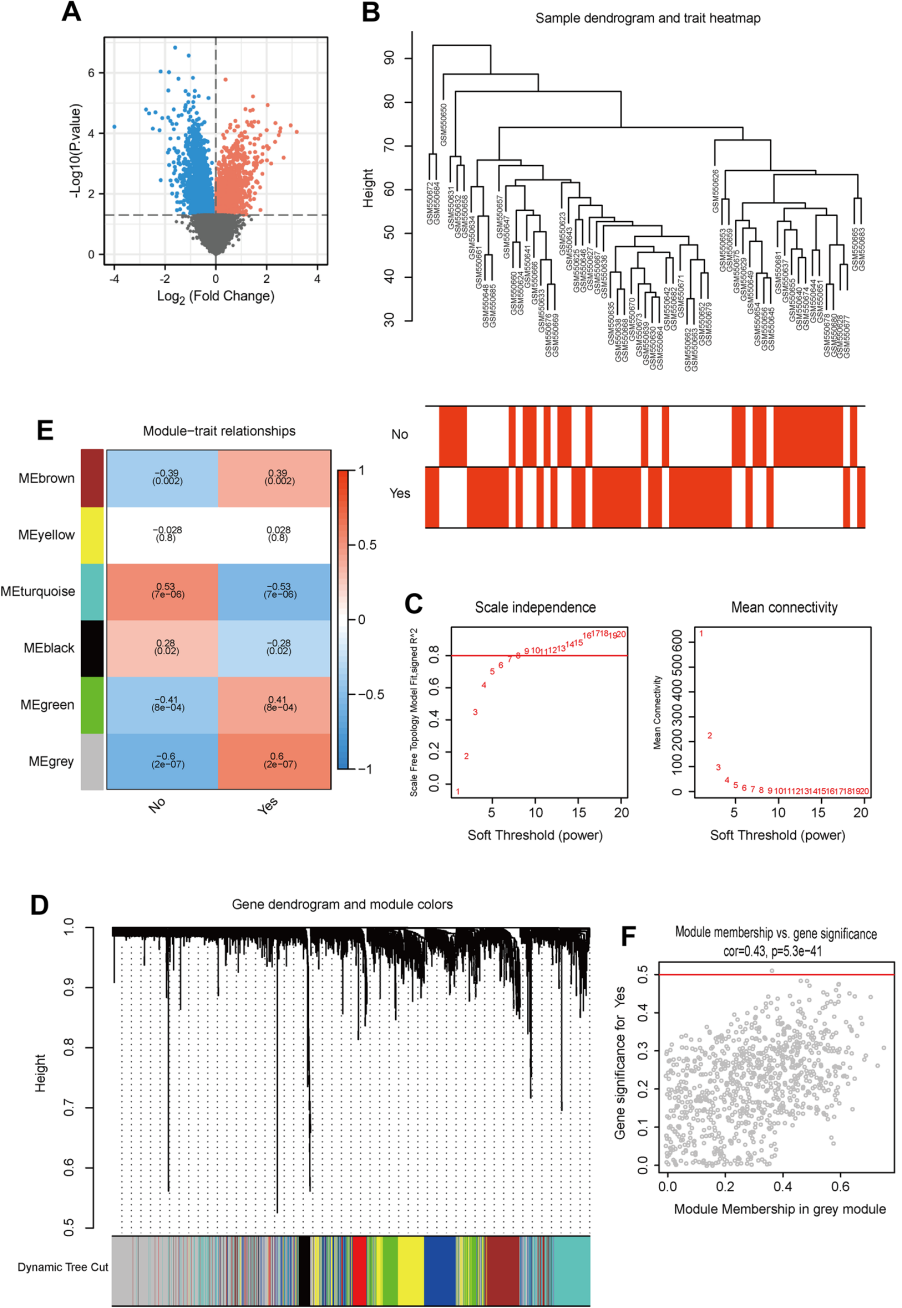
The genes with differential expression between UVM samples with and without metastasis. **A** Volcano Plot exhibited the discrepantly expressed genes in UVM samples with or without metastasis according to GSE22138 dataset (Tumor versus Normal). Red: Up-regulated genes; Blue: Down-regulated genes. **B** The WGCNA was conducted to probe co-expression functional modules related to UVM in GSE22138. **C** The values of the soft threshold (*x*-axis) and the scale-free fit index (*y*-axis) or the mean connectivity (*y*-axis). **D** Clustering dendrogram of genes, with dissimilarity based on topological overlap, together with assigned dynamic tree cut module colors. **E** Module-trait relationships. Row: modules; Columns: status represents the survival status of patients and survival represents the survival time. Each cell contains the matching correlation and *P* value. The table is color-coded by relevance on the basis of the color legend. **F** A scatterplot of gene significance for survival vs. module membership

First, differential expression analysis was performed on the transcription profiles of two groups (metastasis, yes; no metastasis, no) of samples in the GSE22138 database, and 5362 genes with differential expression were obtained, among which 2659 genes were up-regulated in the non-metastasis group and 2703 genes were up-regulated in the metastasis group (Fig. 1A). WGCNA analysis was performed based on this differential gene. To dislodge sample outliers, UVM samples were clustered according to the gene expression matrix, and a clustering tree was established (Fig. 1B), in which the horizontal axis corresponds to each sample, and vertical coordinates represent clustering distances. None of these samples showed a significant deviation, and no samples were removed (Fig. 1C). The selection criterion for the soft threshold was set as signed *R*^2^ > 0.8, and a set of alternative thresholds was selected to export the matching network arguments. As shown in Fig. 1D, when the soft threshold was 7, the gene network exhibited both high internal connectivity and high gene similarity. The gene co-expression network was established with a threshold value of 7, hierarchical clustering of genes with differential expression was performed based on the heterogeneity matrix, and a clustering tree was established (Fig. 1E). The network modules were set to cover a minimum of 50 genes, different gene modules were authenticated by the dynamic cutting method, modules with high similarity were merged, finally, 6 different gene modules were obtained. These different gene modules are represented by different colors, and genes in the same color modules have high similarity. To filter the modules with high associations with UVM metastasis, we first analyzed the principal components of genes in each module; the value of the first principal component was extracted as the module eigenvalue (ME), and then the correlation coefficient between the module eigenvalue and glycolytic type or glutamine decomposition type was calculated. The relevant heat map is shown in Fig. 1F. We found that gray modules were the most relevant to UVM metastasis. The significance and module membership of genes in turquoise modules are shown in Fig. 1E F, and the values of these variables exhibited a strong positive association (cor = 0.43, *P* = 5.3e-41).

### GO analysis and the screening of candidate genes

**Fig. 2:**
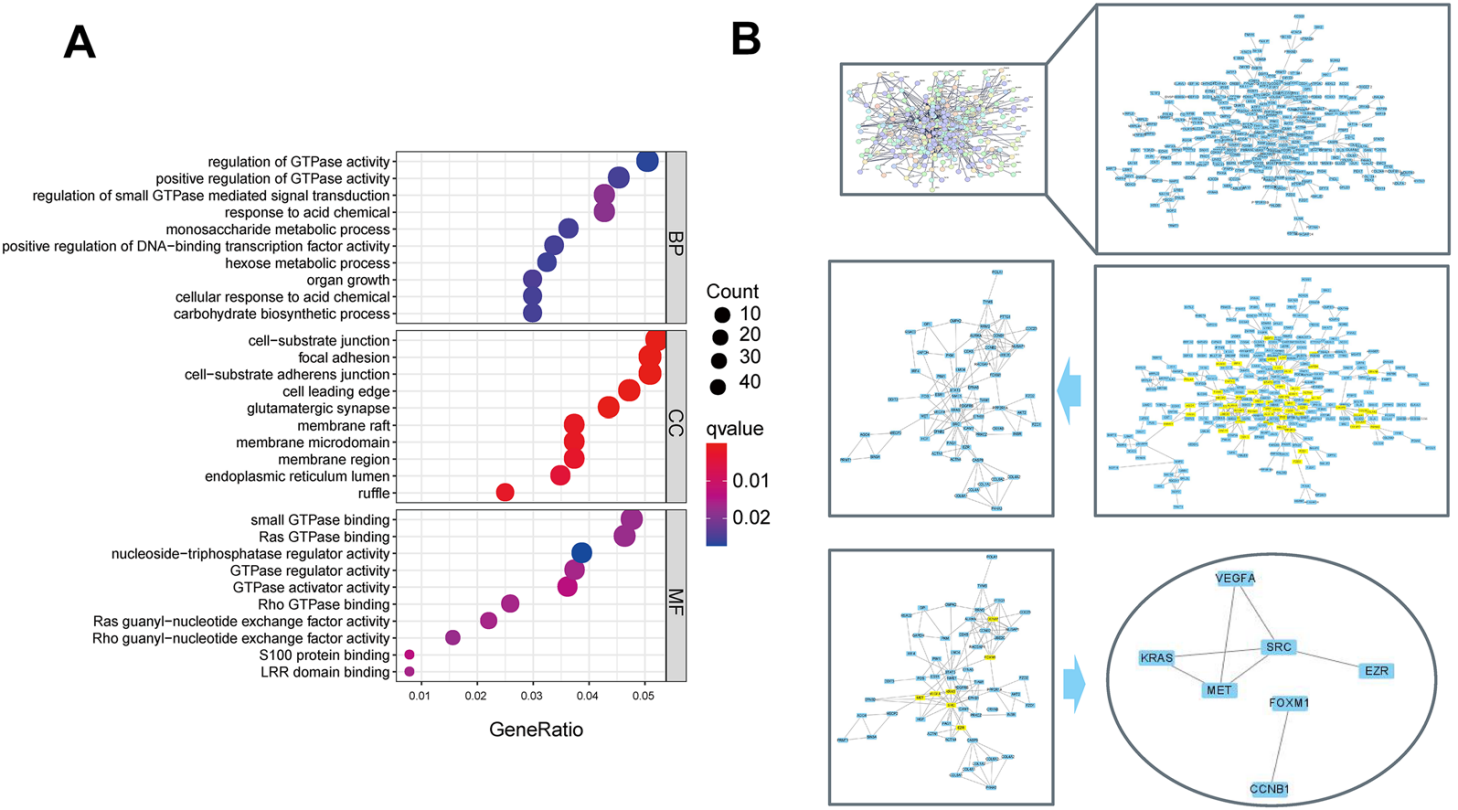
GO enrichment analysis of the hub genes and the screening of candidate genes. **A** Top ranking results of GO enrichment analysis. **B** Topological analysis of PPI network

In view of the above significance, GO enrichment analysis was carried out for the gray modules. A total of 61 items were enriched in GO analysis, including 12 molecular functions, 30 biological processes, and 19 cellular components. Figure 2A shows the top 10 biological processes, molecular functions, and cellular components in which the core target genes are involved. Among them, the biological processes are mainly concentrated in regulating GTPase activity, DNA-binding transcription factor activity, and organ growth; the cell components are mainly concentrated in the cell-substrate junction, focal adhesion, cell leading edge, etc. and are mainly concentrated in Ras GTPase binding, small GTPase binding, nucleoside-triphosphatase regulator activity, etc. To screen out the core genes connected with the UVM further, the aforementioned gray module genes were input into the STRING database, and human genes were selected for protein interaction network analysis (relevant protein nodes were obtained by screening the interactional score ≥ 0.9). To obtain a PPI network diagram, the TSV file of the PPI network was imported into Cytoscape 3.9.0 (Fig. 2B). The CytoNCA plugin was used to topologically analyze the PPI network topologically for 2 times. Seven candidate genes (VEGFA, KRAS, MET, SRC, EZR, FOXM1, and CCNB1) were identified.

### FOXM1 was overexpressed in UVM cells

**Fig. 3:**
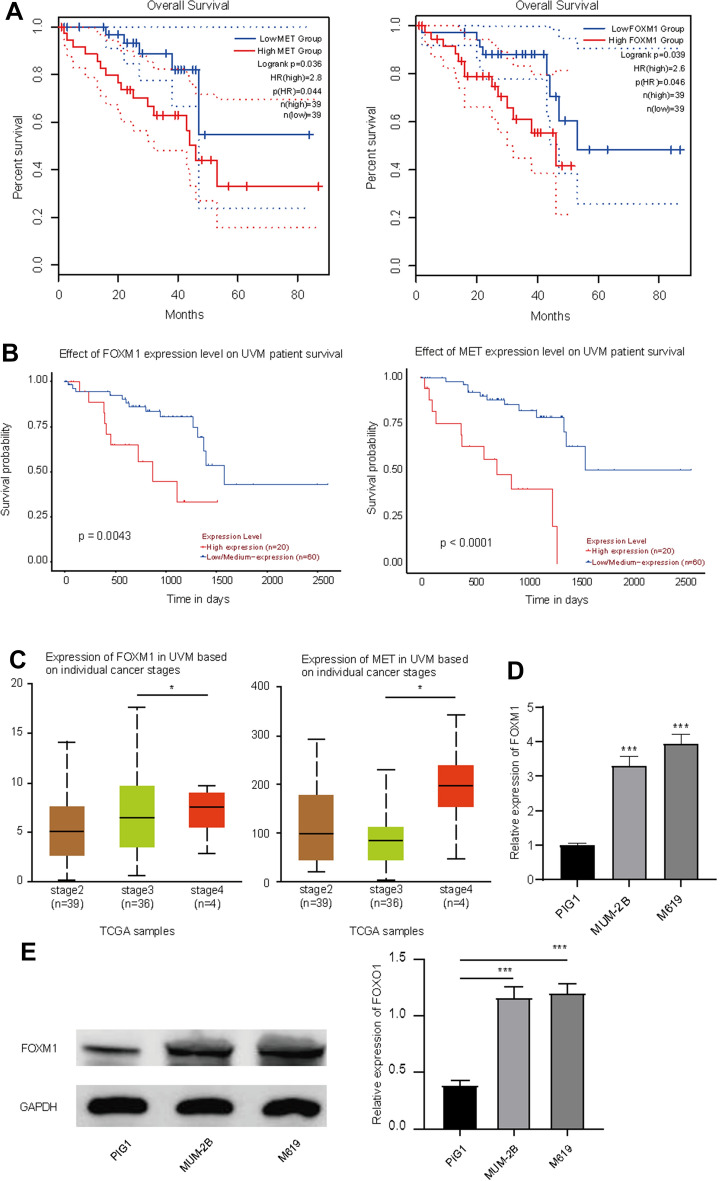
Prognostic analysis of FOXM1 and MET in UVM patients and FOXM1 expression in UVM cells. (**A** and **B**) Kaplan– Meier curve for high-and low-risk groups of UVM patients in GEPIA (**A**) and UALCAN database (**B**). The relationship of individual cancer stages of UVM patients with the expression of FOXM1 and MET in UALCAN database (**C**). The expression of FOXM1 in UVM cells and normal PIG1 cells was tested using qRT-PCR (**D**) and western blot (**E**). ***P < 0.001

Next, the relationship between each of the above-mentioned seven genes and the prognosis of UVM patients (OS) was further explored. Through the analysis of 5-year survival rate of patients with UVM in GEPIA (http://gepia.cancer-pku.cn/index.html) (Fig. 3A) and UALCAN (https://ualcan.path.uab.edu/analysis.html) (Fig. 3B) online databases, it was demonstrated that patients with high FOXM1 or MET expression had poorer overall survival rates, as reflected by Kaplan–Meier analysis (Fig. 3A, B). These results suggest that only two genes (FOXM1 and MET) are associated with the prognosis of UVM patients. Therefore, FOXM1 and MET were identified as core genes associated with UVM metastasis. The TCGA database (https://ualcan.path.uab.edu/cgi-bin/TCGAExResultNew2.pl?genenam=MET&ctype=UVM) analyzed in UALCAN also suggested that individual cancer stages were related to the expression of FOXM1 and MET, in which stage 4 had higher FOXM1 and MET levels than other stages (Fig. 3C). Here, we selected FOXM1 for further studies on UVM pathogenesis. To probe the function of FOXM1 in UVM, the expression of FOXM1 in normal PIG1 and UVM cells was tested. As shown in Fig. 3D, E, the abundance of FOXM1 was notably elevated in UVM cells (MUM-2B and M619) compared to that in normal PIG1 cells. Based on this, we deduced that aberrantly increased levels of FOXM1 might be associated with the advancement and poor prognosis of UVM.

### FOXM1 silence restrained the proliferation, migration and invasion in UVM cells

**Fig. 4:**
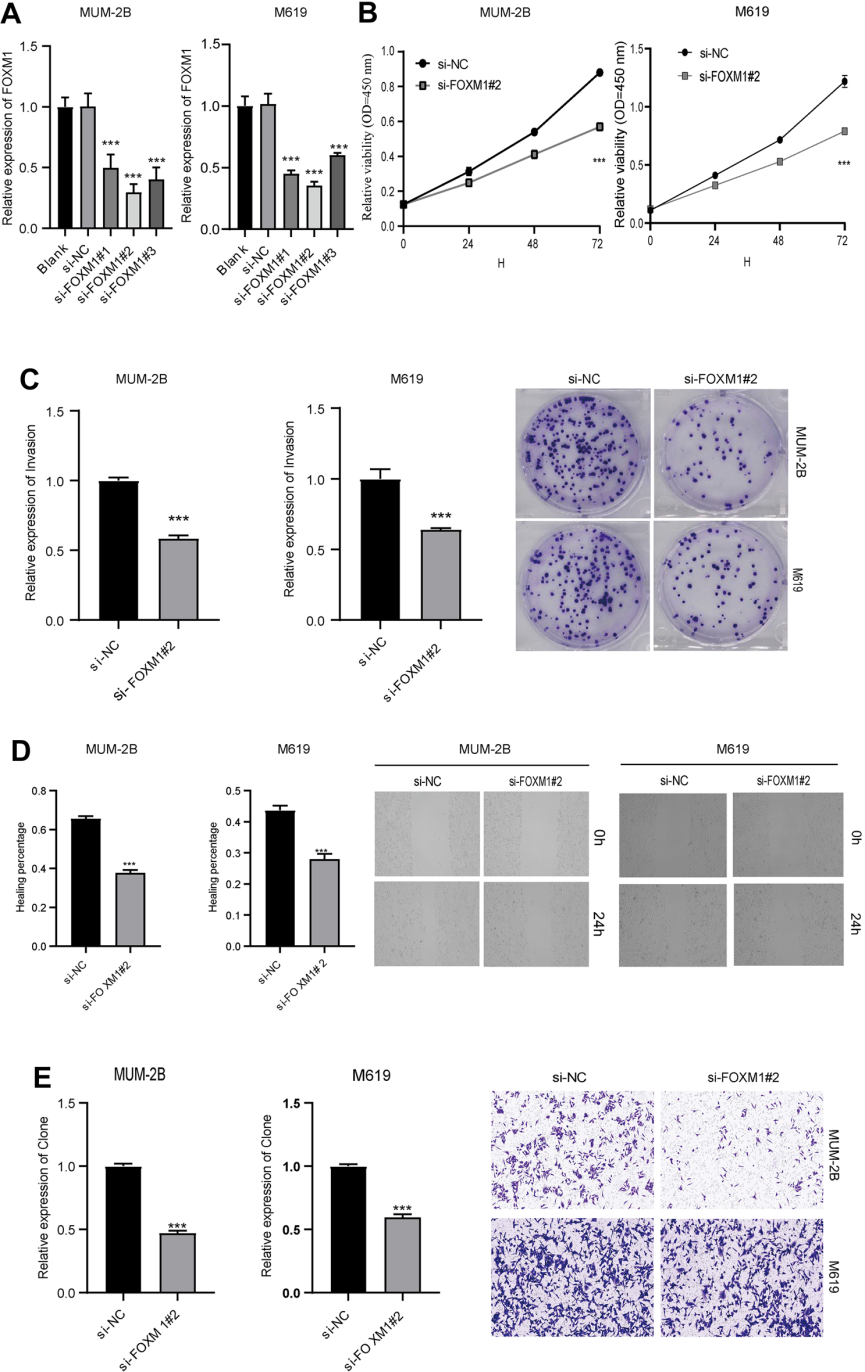
FOXM1 silence hampered cell proliferation, migration and invasion in UVM cells. **A** MUM-2B and M619 cells were introduced with si-NC, si-FOXM1#1, si-FOXM1#2 or si-FOXM1#3, the transfection efficiency of si-FOXM1 was analyzed with qRT-PCR. (**B**–**E**) The si-FOXM1#2 or si-NC was transduced into MUM-2B and M619 cells. **B** The viability of MUM-2B and M619 cells was tested via MTT analysis. **C** The number of MUM-2B and M619 cell colonies was evaluated by cell clone formation assay. **D** The migratory ability of cells was tested using wound healing assay. **E** Transwell assay was employed to estimate the number of invasion cells (× 100). ***P < 0.001

To elucidate the biological function of FOXM1 in UVM cell development, si-NC, si-FOXM1#1, si-FOXM1#2, and si-FOXM1#3 were transduced into MUM-2B and M619 cells. As expected, FOXM1 levels were dramatically reduced in UVM cells after si-FOXM1#1, si-FOXM1#2, or si-FOXM1#3 transfection relative to the control si-NC and non-transfection groups (blank) (Fig. 4A), demonstrating the successful knockdown of FOXM1. Therefore, si-FOXM1#2, with a higher transfection efficiency, was chosen for subsequent experiments. Loss-of-function assays revealed that FOXM1 interference led to an evident impediment in cell viability and clonal numbers of MUM-2B and M619 cells (Fig. 4B, C). Wound healing and Transwell assays confirmed that FOXM1 deficiency overtly restrained MUM-2B and M619 the migratory and invasive abilities (Fig. 4D, E). To summarize, FOXM1 silencing represses the development of UVM cells.

### FOXM1 might affect UVM cell growth by regulating cell cycle arrest

**Fig. 5:**
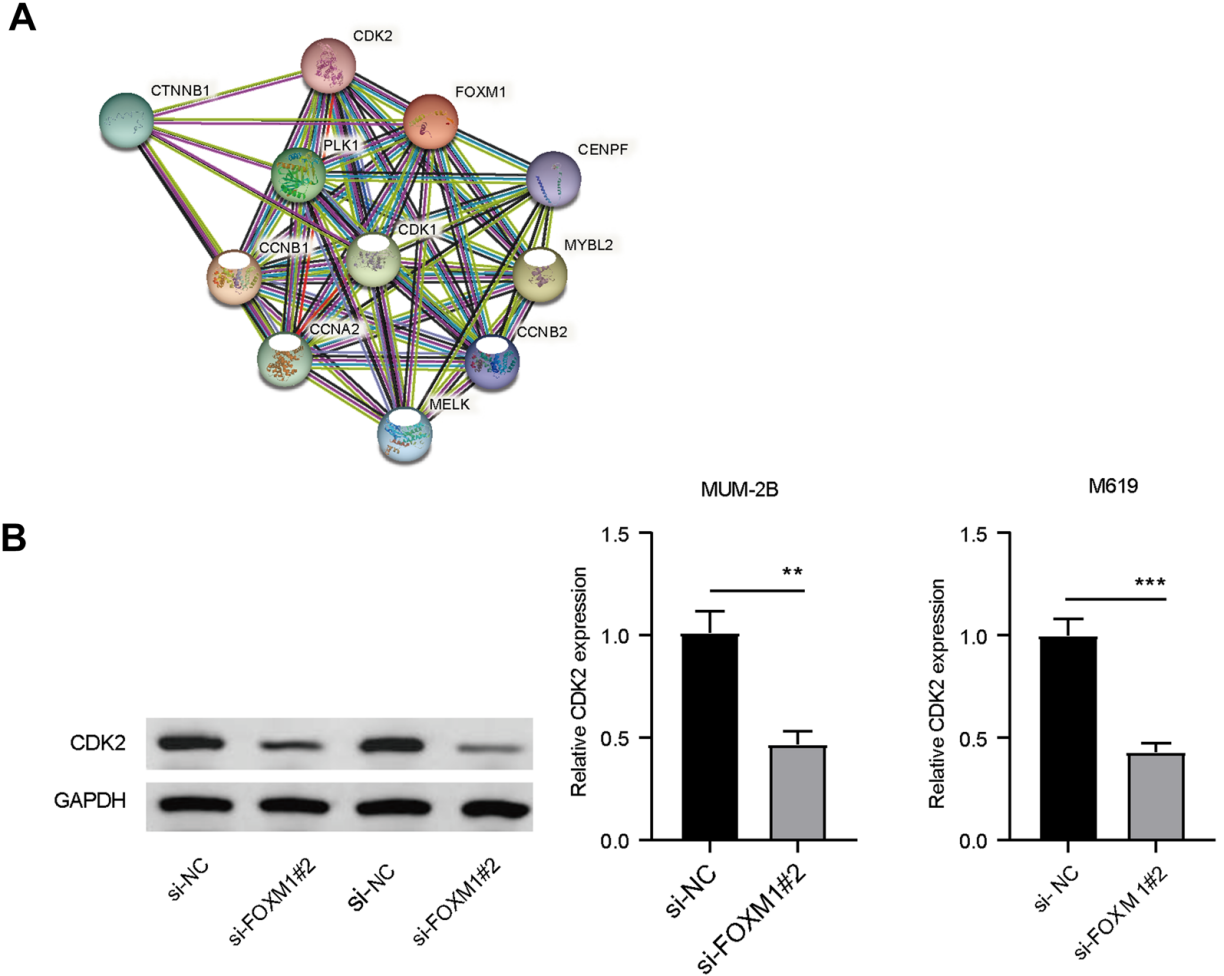
The possible interacting factors of FOXM1 analyzed by bioinformatics online database. **A** The potential interacting protein of FOXM1 using GeneCards. **B** The expression of CDK2 in MUM-2B and M619 cells transfected with si-FOXM1#2 or si-NC was tested by western blot

To probe the regulatory mechanism of FOXM1 in UVM cell development, the potential interacting proteins of FOXM1 were probed. As shown in Fig. 5A, the STRING online network (https://cn.string-db.org/cgi/network?taskId=b450Od5yPz9x&sessionId=bCcb7CZ3idrj) suggested that 10 genes were predicted to interact with FOXM1, some of which were connected to the cell cycle. In addition, through GeneCards analysis (https://www.genecards.org/), FOXM1 was found to participate in several biological processes, including cell cycle, DNA repair, and transcription regulation (Supplementary Table 1). KEGG analysis revealed that FOXM1 participates in cellular senescence (map04218, Supplementary Fig. 1), whereas CDK2 participates in FOXM1-mediated cell cycle arrest. In a subsequent study, we explored the relationship between FOXM1 and CDK2 in UVM cells. As exhibited in Fig. 5B, the expression of CDK2 was declined in MUM-2B and M619 cells transfected with si-FOXM1#2. Therefore, we speculated that FOXM1 may regulate UVM cell cycle arrest by interacting with CDK2.

## Discussion

UVM is the most frequent aggressive intraocular malignancy of the eye in adults [5]. Although the treatment and prognosis of metastatic UVM have improved, there is still a high death rate in UVM patients [15]. Therefore, it is vital to identify potential prognostic markers for UVM treatment. The foregoing studies have offered the framework of co-expression gene modules of UVM and recognized that some prognostic indicators might be utilized for the exploration of relapse and remedies for UVM [16]. Here, we identified differentially expressed genes between UVM samples with or without metastasis using the GEO database (GSE22138 datasets) and further explored the function of FOXM1 in the growth and metastasis of UVM cells.

GEO is an international public repository that archives and freely distributes high-throughput microarray and other functional genomic data sets submitted by the research community [17, 18]. At present, correlation networks are cumulatively utilized in bioinformatics applications. WGCNA can be utilized to identify clusters (modules) of genes with high correlation to identify alternative biological indicators or treated targets [13]. Similarly, co-expression modules were established using WGCNA, and the underlying premonitory indicators of UVM were identified [16]. The differentially expressed mRNAs were confirmed in this study based on the GEO database (GSE22138). WGCNA was used to explore co-expression of functional modules related to UVM in GSE22138. Through topological analysis of the PPI network, seven candidate genes (VEGFA, KRAS, MET, SRC, EZR, FOXM1, and CCNB1) were identified. By analyzing the relationship between these seven genes and the prognosis of UVM patients, only two genes (FOXM1 and MET) were found to be associated with poor prognosis in UVM patients. Hence, FOXM1 and MET were identified as the core genes associated with UVM metastasis. Through UALCAN analysis, high expression of FOXM1 or MET was related to shorter overall survival of UVM patients and higher cancer stages of UVM. Here, we selected FOXM1 for further studies on UVM pathogenesis.

Previous studies have demonstrated the function of FOXM1 in various cancers [19, 20]. Nevertheless, the role of FOXM1 in UVM prognosis prediction and etiology of UVM metastasis is still restricted and requires further investigation. Previous studies have proposed that FOXM1 is up-regulated in cutaneous melanoma [12, 21]. In addition, consistent with the GSE22138 dataset, FOXM1 was dramatically up-regulated in UVM cells (MUM-2B and M619) compared to that in normal PIG1 cells. In addition, FOXM1 downregulation caused obvious repression of UVM cell growth and metastasis. Thus, based on the above findings, we deduced that FOXM1 may exert a carcinogenic effect on UVM progression. Later, the regulatory mechanism of FOXM1 in UVM cells was further explored. By using STRING online network, 10 cell cycle-related genes were predicted to interact with FOXM1, and GeneCards analysis suggested that FOXM1 participated in several biological processes, including cell cycle, DNA repair, and transcription regulation. Meanwhile, KEGG analysis revealed that CDK2 participates in FOXM1-mediated cell cycle arrest in map04218. Precious studies have suggested that FOXM1 overexpression was associated with tumor progression in patients with clear cell renal cell carcinoma [22], and FOXM1 silence could lead to liver cancer cell growth and a decline of CDK2 expression [23]. Therein, whether CDK2 was involved in FOXM1-mediated UVM cell development was further explored. The result suggested that CDK2 was down-regulated in MUM-2B and M619 cells after FOXM1 silence. Thus, we deduced that FOXM1 might regulate UVM cell cycle arrest by interacting with CDK2.

In conclusion, our current study revealed that FOXM1 was elevated in UVM, and FOXM1 deficiency could efficiently block the vicious advancement of UVM. This study may provide a promising curative target for UVM patients and serve as a reference for understanding other cancers.

## Supplementary Information

Below is the link to the electronic supplementary material.

Supplementary file 1 (DOCX 16 kb)

Supplementary file 2 (TIF 200 kb)

## References

[1] Rastrelli M et al (2014) Melanoma: epidemiology, risk factors, pathogenesis, diagnosis and classification. In Vivo 28(6):1005–1011 25398793

[2] Slominski A et al (2001) Malignant melanoma. Arch Pathol Lab Med 125(10):1295–1306 11570904 10.5858/2001-125-1295-MM

[3] Ortega MA et al (2020) Update on uveal melanoma: translational research from biology to clinical practice (review). Int J Oncol 57(6):1262–1279 33173970 10.3892/ijo.2020.5140 PMC7646582

[4] Spagnolo F, Caltabiano G, Queirolo P (2012) Uveal melanoma. Cancer Treat Rev 38(5):549–553 22270078 10.1016/j.ctrv.2012.01.002

[5] Kaliki S, Shields CL (2017) Uveal melanoma: relatively rare but deadly cancer. Eye 31(2):241–257 27911450 10.1038/eye.2016.275 PMC5306463

[6] Riechardt AI, Kilic E, Joussen AM (2021) The genetics of uveal melanoma: overview and clinical relevance. Klin Monbl Augenheilkd 238(7):773–780 34376007 10.1055/a-1513-0789

[7] Wierstra I, Alves J (2007) FOXM1, a typical proliferation-associated transcription factor. Biol Chem 388(12):1257–1274 18020943 10.1515/BC.2007.159

[8] Zona S et al (2014) FOXM1: an emerging master regulator of DNA damage response and genotoxic agent resistance. Biochim Biophys Acta 1839(11):1316–1322 25287128 10.1016/j.bbagrm.2014.09.016 PMC4316173

[9] Borhani S, Gartel AL (2020) FOXM1: a potential therapeutic target in human solid cancers. Expert Opin Ther Targets 24(3):205–217 32067537 10.1080/14728222.2020.1727888

[10] Khan MA et al (2023) FOXM1: a small fox that makes more tracks for cancer progression and metastasis. Semin Cancer Biol 92:1–15 36958703 10.1016/j.semcancer.2023.03.007 PMC10199453

[11] Ito T et al (2016) Prognostic significance of forkhead box M1 (FoxM1) expression and antitumour effect of FoxM1 inhibition in melanoma. Histopathology 69(1):63–71 26619071 10.1111/his.12909

[12] Miyashita A et al (2015) Investigation of FOXM1 as a potential new target for melanoma. PLoS ONE 10(12):e0144241 26640950 10.1371/journal.pone.0144241 PMC4671728

[13] Langfelder P, Horvath S (2008) WGCNA: an R package for weighted correlation network analysis. BMC Bioinform 9:559 10.1186/1471-2105-9-559 PMC2631488 19114008

[14] Robertson AG et al (2017) Integrative analysis identifies four molecular and clinical subsets in uveal melanoma. Cancer Cell 32(2):204–220 28810145 10.1016/j.ccell.2017.07.003 PMC5619925

[15] Reichstein D et al (2022) Treatment of metastatic uveal melanoma in 2022: improved treatment regimens and improved prognosis. Curr Opin Ophthalmol 33(6):585–590 36094043 10.1097/ICU.0000000000000905

[16] Wan Q et al (2018) Co-expression modules construction by WGCNA and identify potential prognostic markers of uveal melanoma. Exp Eye Res 166:13–20 29031853 10.1016/j.exer.2017.10.007

[17] Barrett T, Edgar R (2006) Gene expression omnibus: microarray data storage, submission, retrieval, and analysis. Methods Enzymol 411:352–369 16939800 10.1016/S0076-6879(06)11019-8 PMC1619900

[18] Barrett T et al (2013) NCBI GEO: archive for functional genomics data sets—update. Nucleic Acids Res 41(Database issuse):D991-995 23193258 10.1093/nar/gks1193 PMC3531084

[19] Halasi M, Gartel AL (2013) Targeting FOXM1 in cancer. Biochem Pharmacol 85(5):644–652 23103567 10.1016/j.bcp.2012.10.013

[20] Liao GB et al (2018) Regulation of the master regulator FOXM1 in cancer. Cell Commun Signal 16(1):57 30208972 10.1186/s12964-018-0266-6 PMC6134757

[21] Sun M et al (2017) MicroRNA-216b inhibits cell proliferation and migration in human melanoma by targeting FOXM1 in vitro and in vivo. Cell Biol Int 41(12):1272–1282 28225180 10.1002/cbin.10754

[22] Xue YJ et al (2012) Overexpression of FoxM1 is associated with tumor progression in patients with clear cell renal cell carcinoma. J Transl Med 10:200 23006512 10.1186/1479-5876-10-200 PMC3492118

[23] Wu CH, Yeh CT, Lin KH (2020) Thyroid hormones suppress FOXM1 expression to reduce liver cancer progression. Oncol Rep 44(4):1686–1698 32945493 10.3892/or.2020.7716

