# Supplementary material for: FOXM1 promote the growth and metastasis of uveal melanoma cells by regulating CDK2 expression
Source: Int Ophthalmol. 2024 Feb 11;44(1):55. doi: 10.1007/s10792-024-02943-y (PMC10859341; doi:10.1007/s10792-024-02943-y)
Supplement: Supplementary file 1 — (DOCX 16 kb) [file 10792_2024_2943_MOESM1_ESM.docx]

| **GO ID** | **Qualified GO term** |
| --- | --- |
| GO:000008635 | involved_in G2/M transition of mitotic cell cycle |
| GO:000012235 | involved_in negative regulation of transcription by RNA polymerase II |
| GO:00015585 | regulation of cell growth |
| GO:000628135 | involved_in DNA repair |
| GO:00063555 | regulation of DNA-templated transcription |
| GO:00063575 | regulation of transcription by RNA polymerase II |
| GO:00069745 | cellular response to DNA damage stimulus |
| GO:000697835 | involved_in DNA damage response, signal transduction by p53 class mediator resulting in transcription of p21 class mediator |
| GO:00070495 | cell cycle |
| GO:000828435 | involved_in positive regulation of cell population proliferation |
| GO:003287335 | involved_in negative regulation of stress-activated MAPK cascade |
| GO:00421275 | regulation of cell population proliferation |
| GO:004589235 | involved_in negative regulation of DNA-templated transcription |
| GO:004589335 | involved_in positive regulation of DNA-templated transcription |
| GO:004594435 | involved_in positive regulation of transcription by RNA polymerase II |
| GO:004657835 | involved_in regulation of Ras protein signal transduction |
| GO:005172635 | acts_upstream_of regulation of cell cycle |
| GO:200037735 | involved_in regulation of reactive oxygen species metabolic process |
| GO:200078135 | involved_in positive regulation of double-strand break repair |

**Table 1. The exhibition of biological processes for FOXM1 by GeneCards**
