# Supplementary figures and images for: FOXM1 promote the growth and metastasis of uveal melanoma cells by regulating CDK2 expression
Source: Int Ophthalmol. 2024 Feb 11;44(1):55. doi: 10.1007/s10792-024-02943-y (PMC10859341; doi:10.1007/s10792-024-02943-y)

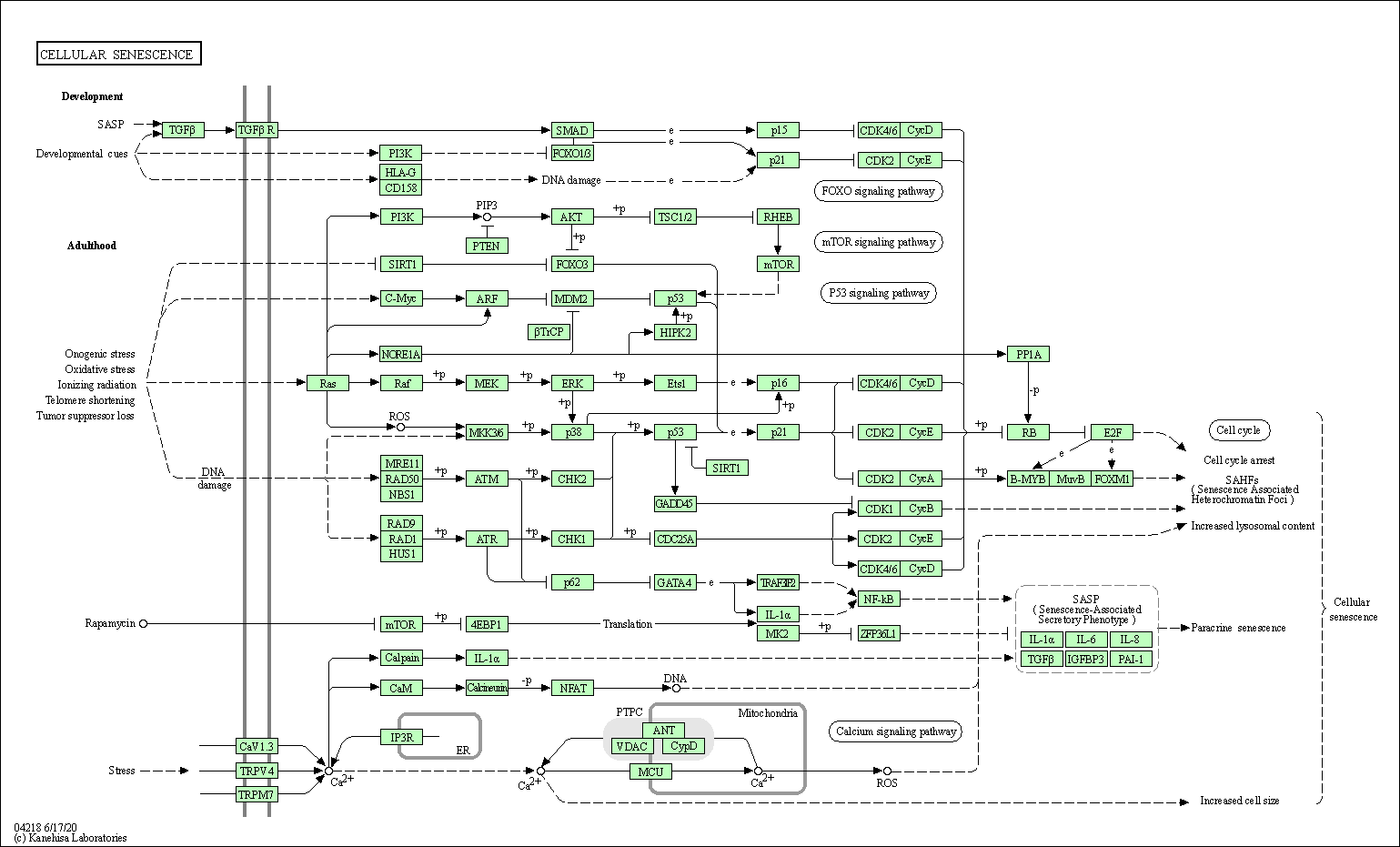

Supplement: Supplementary file 2 — (TIF 200 kb) [file 10792_2024_2943_MOESM2_ESM.tif]
